# Determinants of Outcomes for Acute Myeloid Leukemia Patients Treated in a Community-Based Specialized Versus Non-Specialized Hospital Setting

**DOI:** 10.46989/001c.124273

**Published:** 2024-10-09

**Authors:** Minoo Battiwalla, Ju-Hsien Chao, Tonya Cox, Jose Carlos Cruz, William B. Donnellan, Alireza Eghtedar, Suman Kambhampati, Shahbaz Malik, Michael B. Maris, Marcello Rotta, Frank T. Slovick, Aravind Ramakrishnan, Vikas Bhushan, Lindsay Sears, Casey Martin, Jared Holder, Angela Junglen, Navneet Majhail, Charles F. LeMaistre

**Affiliations:** 1 Sarah Cannon Blood Cancer Network; 2 Sarah Cannon Center for Blood Cancer at TriStar Centennial; 3 Texas Transplant Institute; 4 Tennessee Oncology; 5 Colorado Blood Cancer Institute; 6 HCA Midwest Health at Research Medical Center; 7 St. David’s South Austin Medical Center; 8 Medical City Dallas Hospital; 9 Healthcare Corporations of America Research Institute

**Keywords:** AML, real-world evidence, center effects, community-based

## Abstract

The treatment setting influences acute myeloid leukemia (AML) outcomes. Most cancer patients receive care in the community, yet few studies have evaluated the effectiveness of clinical programs outside of academic or National Cancer Institute (NCI)-designated cancer centers. This was a multi-level, case-controlled study of real-world outcomes for initial AML treatment in a community-based network for 1,391 patients with AML between 2011 and 2018. We benchmarked survival within our network against the Surveillance, Epidemiology, and End Results (SEER) database. Coarsened exact matching was performed against 17,186 chemotherapy-treated patients in the SEER database. Cox proportional and accelerated failure time multivariable modeling were performed to identify patient, disease, therapy and center characteristics associated with the risk of AML mortality. Within the network, 799 patients were treated at six specialized blood cancer centers and 592 at 63 other hospitals. Patients receiving high-intensity induction at specialized centers had improved median survivals of 31 months versus 18 months [P<0.001] at non-specialized centers. Median survivals were 13 for non-specialized centers versus 10 months for SEER [P<0.001], and 18 for the entire network versus 10 months for SEER [P<0.001]. Multivariable modeling showed significant impacts from age (*HR* = 1.025), high-intensity induction therapy (*HR*= .695), diagnosis year (*HR*= .937), neighborhood income (*HR* = .997; P<0.01), higher acuity (*HR* = 1.002) and Charlson comorbidity score (*HR* = 1.117).

AML treatment may be effectively delivered in the community hospital setting, with specialized centers producing better outcomes for higher intensity treatments.

## Introduction

Acute myeloid leukemia (AML) is a group of heterogeneous malignancies characterized by clonal expansion and arrest at the blast stage of a malignant hematopoietic precursor, which leads to lethal complications if patients are not promptly evaluated and treated. The clinical course of AML is often complicated requiring intensive support.

Understanding the outcomes for AML therapy requires an appreciation of the treatment setting. Clinical trials for AML generally select for younger and fitter patients, in a disease where the median age of onset is 68 years,^1^ and where the disease acuity and treatment tolerance are determined by the patient’s age.^2,3^ Patients with AML reported from academic centers tend to be younger, with fewer co-morbidities, and more likely to receive aggressive therapy, such as intensive chemotherapy and transplantation.^4–6^ Socio-economic barriers clearly predict outcomes.^7,8^ Most cancer care takes place at the community level,^9^ and generating real-world evidence for AML outcomes by studying unselected populations in this setting is valuable.

As with other complex therapies, treatment of AML varies among centers, including patient selection for aggressive therapy, supportive care practices and management of disease and treatment-related complications.^10^ Additionally, infrastructure and care delivery models may vary among centers. Variation in center practices, experience and resources may influence patient outcomes. While treatment at specialized centers is desirable, this is not always possible, and the vast majority of cancer patients (including those with leukemia) are treated in the community.^4,11^ Identification of center characteristics that improve survival is necessary to improve care delivery in the community setting.

Few studies have examined the association of center characteristics and survival of AML. Previous research has demonstrated that early mortality and complications for AML patients treated at National Cancer Institute (NCI) Comprehensive Cancer Centers (NCICCC) were significantly lower compared to non-NCI Cancer Centers in California.^12^ That study, however, did not investigate the reasons for these center-level differences in patient outcomes. An earlier study demonstrated that while survival of patients with AML in North Carolina varied with geographic region, treatment at a NCICCC was not associated with reduced mortality at one year.^13^ The authors suggested that NCICCC designation may not be an appropriate indicator of quality in this patient population. AML patient survival has been linked to center volume, clinical trial access, and the presence of multidisciplinary team meetings.^10,14,15^

In this study, we examined real-world AML outcomes in a large network of 185 community hospitals (HCA Healthcare (HCA)) between 2011 and 2018. Within the HCA community network, we also identified patients treated at the Sarah Cannon Blood Cancer Network (SCBCN) comprising six high-volume hospitals that have been certified as meeting defined metrics. HCA Healthcare attempts to concentrate leukemia care to these specialized centers with infrastructure, appropriate staffing, use of standardized protocols and processes, and the expertise and resources to provide that care. We then examined patient, disease and center-related characteristics influencing outcomes in the community network and validated them against those reported by the Surveillance, Epidemiology, and End Results (SEER) database.

The objectives of the current study were to:

1. Compare overall survival of patients receiving high intensity induction therapy at the SCBCN specialized centers to those at other HCA Healthcare community-based cancer care centers (non-SCBCN).
2. Compare overall survival of patients at community hospitals without specialized cancer centers to the benchmark of chemotherapy-treated SEER patients (non-SCBCN versus SEER comparison).
3. Compare the overall survival of chemotherapy-treated patients receiving care at HCA Healthcare community hospitals to SEER patients (HCA Healthcare versus SEER Comparison).
4. Identify patient, disease, therapy, and center characteristics associated with the mortality risk.

## Methods

### Data Acquisition

Data were collected from HCA Healthcare’s cancer registry and electronic medical records and from the most recent version of the SEER dataset, which includes chemotherapy treatment information from the NCI.^1^ These represent real-world observational data gathered during the delivery of standard-of-care treatment in the community setting.

### Patient Inclusion/Exclusion

**Figure 1.**
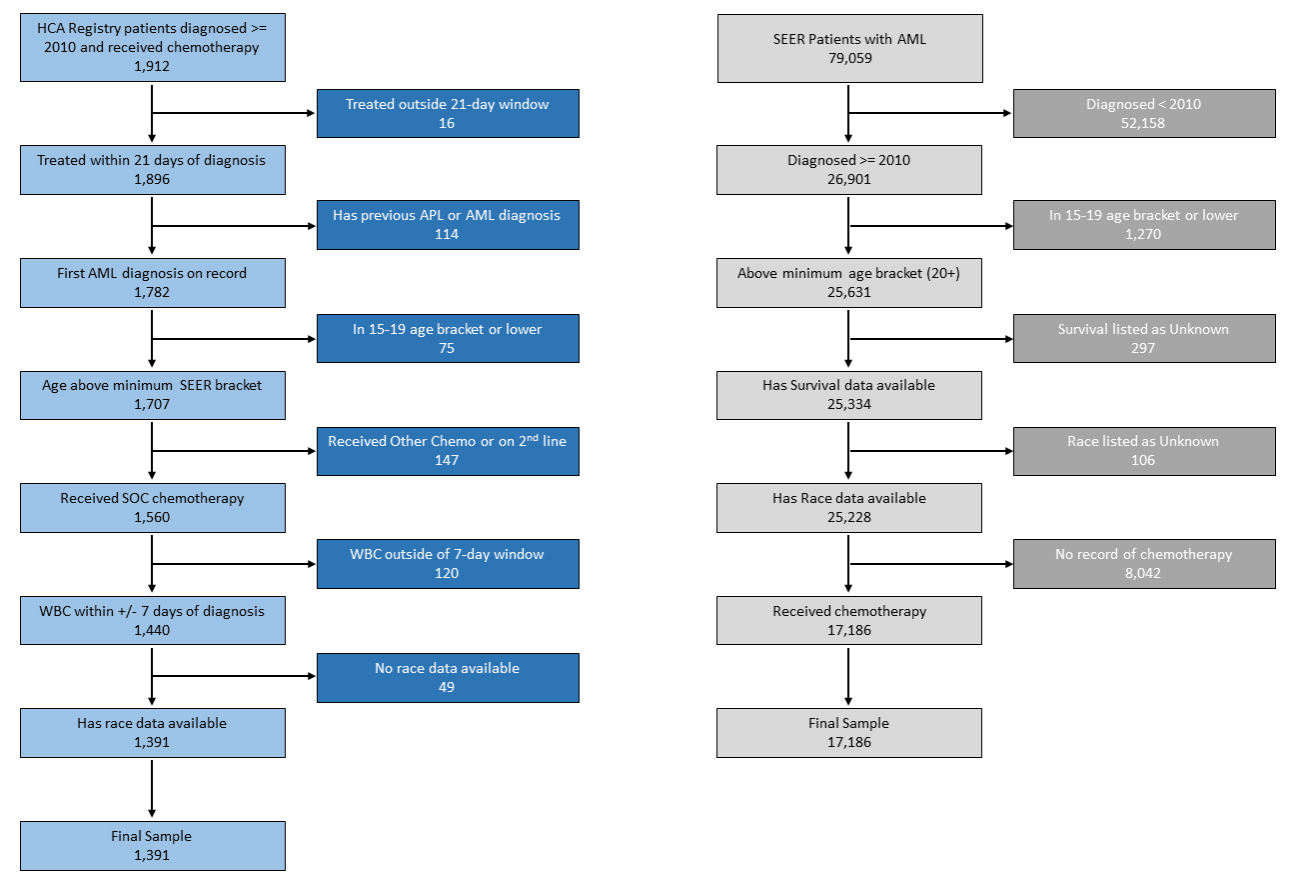
selection for the final sample for both HCA Healthcare and SEER data

The selection for the final sample for both HCA Healthcare and SEER data is summarized in a filtering flow chart (Figure 1). HCA Healthcare patients were included in the samples if they were at least 18 years old, had a primary diagnosis of AML, had accessible medical records, were hospitalized for treatment with cytarabine with or without anthracycline, or with hypomethylating agents for their induction therapy. The SEER sample consisted of a minimum age of 20 years, a primary diagnosis of AML, and an indication of chemotherapy. All AML diagnoses were determined using ICD-O-3 histology codes (see Supplemental Tables). Acute promyelocytic leukemia (APL) was excluded. For comparisons between SEER and HCA Healthcare cohorts, the latter was restricted to patients aged 20 years or older in order to correspond to SEER’s minimum age for defining adults (ages 20-24 years).

SEER began data collection in 1973. In order to aid matching with more recently established HCA Healthcare data sources, both datasets were limited to years 2010 onwards. While the primary drugs used in the standard of care in AML, cytarabine and anthracyclines, have not changed since the 1970s, supportive care has markedly improved in the past few decades and patient survival is significantly higher in recent years.^16^

Within the SEER dataset, the final sample size for our comparisons was 17,186. Within the HCA Healthcare dataset, it was 1,391, which consisted of 799 patients treated at a SCBCN center and 592 patients at a non-SCBCN facilities.

### Measures

For HCA Healthcare-to-SEER comparisons, the analyses were limited to the data fields available in the SEER dataset, which are vital status, months survived until death or censorship, age bin at diagnosis, race, and gender. Race was categorized as White, Black or other due to low volumes for other groups. Months survived was calculated as the number of full months survived up until the last contact date. Patients were not censored for hematopoietic stem cell transplantation as this indicator is not available in the SEER dataset.

To examine additional patient and center characteristics, the following variables were collected in the HCA Healthcare dataset: daily survival, mean income based on patient home zip code, acuity at diagnosis, patient comorbidities, treatment center zip code, and whether the center was part of the SCBCN.

Acuity at diagnosis was based on the WBC measurement closest to the diagnosis date within +/- 7 days of diagnosis. In cases where more than one WBC measurement was taken on the target date, the highest WBC measurement was used. If a WBC measurement was not available within the +/-7 day timeframe, the patient was removed from analysis. Patient comorbidities were captured using ICD9 and ICD10 codes available in the patient’s medical record and summarized using the Charlson Comorbidity Index (CCI).^17^ Socioeconomic status was estimated for each patient by assigning the mean income for the patient’s zip code of residence. Mean incomes for patient zip codes were obtained from the Internal Revenue Service.^18^ The distance of the patient’s residence to the treatment center was approximated by acquiring the geographical coordinates for the centroid of the patient’s residential zip code and the zip code of the treatment center. Distance was calculated using Vincenty’s Formula for oblate spheroids in the R package ‘geosphere’.

Center effect variables were examined, including SCBCN status, patient distance from facility, facility bed count, AML treatment volumes, and Commission on Cancer (CoC) accreditation. All SCBCN centers are in large hospitals, treat high volumes of AML patients, and are CoC accredited, resulting in little to no variability within the SCBCN grouping. Due to the high multicollinearity between facility bed count, AML treatment volume, and CoC accreditation, these variables were removed from the analysis.

High dose cytarabine (HiDAC) or cytarabine combined with an anthracycline (“7+3”) were classified as high-intensity induction chemotherapy, and hypomethylating agents, such as decitabine or azacytidine, were classified as low intensity.

### Coarsened Exact Matching

Coarsened exact matching (CEM) was used to control for as several potential confounders as possible when comparing across the following sample sets^19^: HCA Healthcare to SEER, non-SCBCN centers to SEER, and SCBCN centers to non-SCBCN centers within the HCA Healthcare network. A one-to-many matching algorithm was used, with patient-level weights being generated for comparison patients and applied in all subsequent analyses. All groups were matched based on age at diagnosis using five-year bins, gender, and race, and year of diagnosis. Due to the lack of SEER data past 2016, HCA Healthcare patients from 2016-2019 were bucketed and matched to the 2016 diagnosis SEER group.

### Analysis

Our initial analysis examined differences in survival rates for AML patients between HCA Healthcare subsets SCBCN and non-SCBCN, between the non-SCBCN and SEER, and between HCA Healthcare and SEER. CEM was implemented separately for each comparison. We also investigated the effects of other covariates, within the HCA Healthcare network, using a Cox proportional hazards regression model and an accelerated failure time model (when Cox proportional hazards model assumptions were violated). These were implemented to investigate differences in outcomes based on center, treatment and patient characteristics.

Data cleaning and analysis were done using R v4.2. Coarsened exact matching was performed using the “cem” package. Survival analyses (including Cox Proportional Hazards and Accelerated Failure Time) were conducted using the “survival” package.

## Results

### Patient Characteristics

Pre-match patient characteristics stratified by SEER, SCBCN, and HCA Healthcare affiliations are summarized in Table 1. Generally, across the three samples, there were higher percentages of males (53.3-56.3%), majority white (81.4-87.9%) and the highest percentages within the 65-69 age bin (14.3-14.7%). SCBCN patients had similar acuity but were younger, had lower comorbidity burden, higher neighborhood income, and traveled a longer distance than non-SCBCN patients.

**Table 1.** Pre-Match patient characteristics stratified by HCA Healthcare (SCBCN), HCA Healthcare (non-SCBCN) and SEER.

	HCA Healthcare (SCBCN) N = 799 N_centers_ = 6	HCA Healthcare (non-SCBCN) N = 592 N_centers_ = 63	SEER N = 17,186 --
Sex, *N (%)*			
*Male*	426 (53.3%)	323 (54.6%)	9,671 (56.3%)
*Female*	373 (46.7%)	269 (45.4%)	7,515 (43.7%)
Race*,* *N (%)*			
*White*	724 (87.9%)	487 (81.4%)	14,150 (82.1%)
*Black*	31 (3.7%)	76 (12.8%)	1,548 (9.0%)
*Other*	44 (5.3%)	29 (4.8%)	1,488 (8.6%)
Ethnicity*,* *N (%)*			
*Hispanic*	111 (13.9%)	91 (15.4%)	2,061 (12.1%)
*Non-Hispanic*	590 (73.8%)	432 (73.0%)	15,125 (87.9%)
*Unspecified*	98 (12.3%)	69 (11.7%)	--
Age at Diagnosis*, M(SD)*	56.8 (15.7)	62.1 (14.6)	65-69^a^
Therapy Intensity*,* *N (%)*			
*High*	660 (82.6%)	479 (80.9%)	--
*Low*	139 (17.4%)	113 (19.1%)	--
Acuity*, M(SD)*	46.0 (77.6)	45.2 (63.5)	--
Comorbidity Index*, M(SD)*	1.3 (1.8)	1.7 (2.0)	--
Neighborhood income*, M(SD)*	72,859.5 (37,825.8)	63,759.4 (41,5731.0)	--
Distance from center in km*, M(SD)*	91.7 (184.6)	49.1 (187.9)	--
Facility AML Volume*,* *M(SD)*	135.0 (74.5)	9.4 (10.0)	--
Facility CoC Accreditation*,* *N (%)*	6 (100%)	38 (60.3%)	--
Facility Bed Count*,* *M(SD)*	552.7 (205.3)	312.2 (115.4)	--

^a^ Patient ages are not provided by SEER, therefore the median age bin is provided.

### Overall Survival Comparisons

After matching, Kaplan-Meier survival curves were generated. The *L_1_* statistic was assessed for adequacy of matching. Despite smaller sample sizes, matching for SCBCN and non-SCBCN resulted in a reasonable *L_1_* of 0.069. *L_1_* ≈ 0 for both comparisons, with the SEER dataset demonstrating excellent balance across groups.

**Figure 2.**
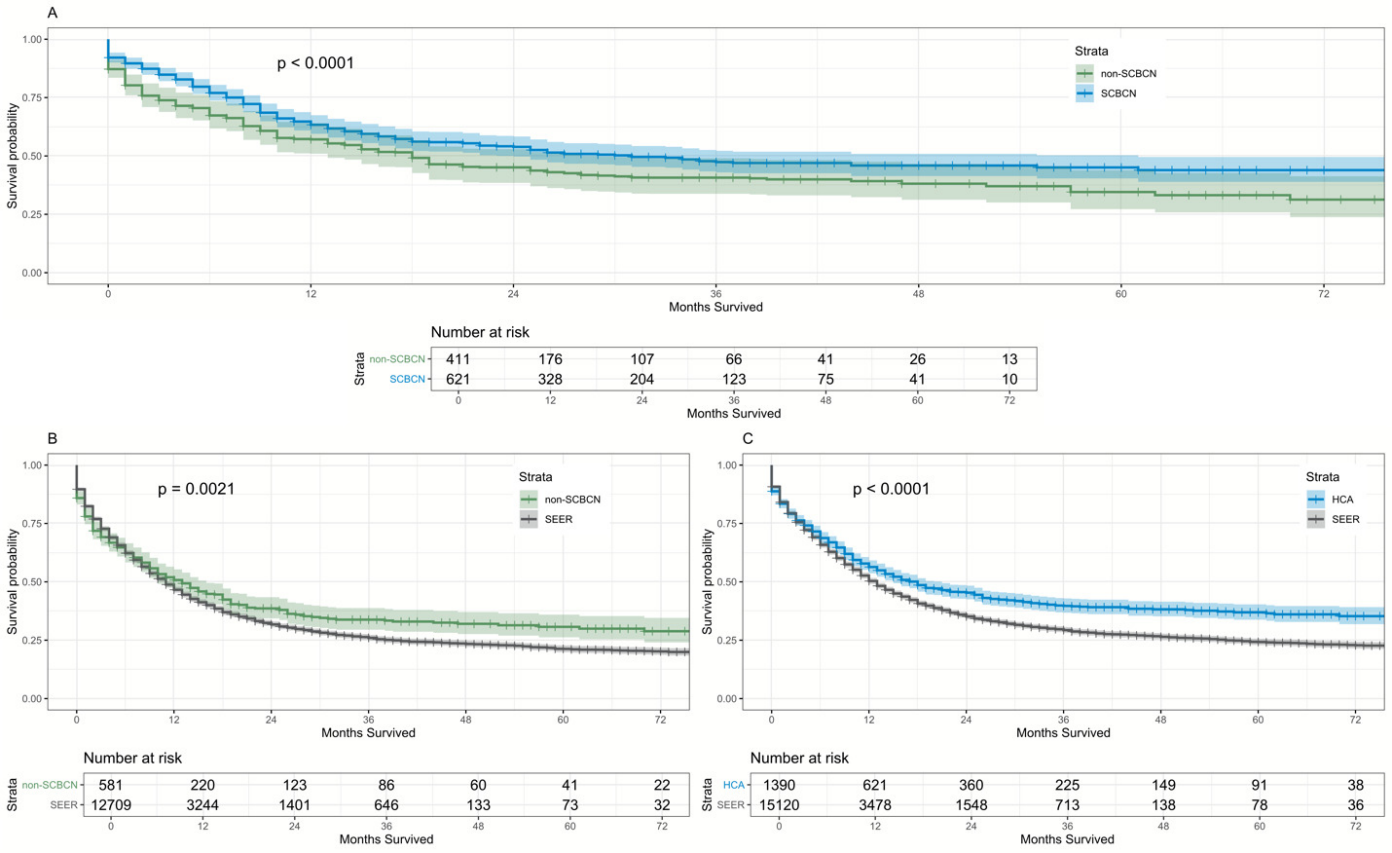
Comparing SCBCN versus non-SCBCN specifically for high-intensity induction therapy.

We compared SCBCN versus non-SCBCN specifically for high intensity induction therapy. After matching, the Kaplan-Meier curves and log-rank test demonstrated a significantly higher overall survival for the 614 SCBCN in comparison to the 422 non-SCBCN patients going through high intensity induction therapy (median 31 months versus 18 months, *p* < .0001; Figure 2A).

For the non-SCBCN (n = 592) to SEER (n = 12,427) matched comparison, results indicated non-SCBCN having better overall survival than SEER (median 13 months versus 10 months, *p* = .0021; Figure 2B).

Comparing HCA Healthcare (n= 1,390) to chemotherapy-treated SEER patients (n= 15,120), those in the former group had significantly higher survival rates than those in the latter (median 18 months versus 10 months, *p* < 0.001; Figure 2C).

### Determinants of survival

Within the HCA Healthcare cohort, we evaluated the following patient and center predictors for their association with survival: age at diagnosis, treatment intensity, diagnosis year, gender, race, CCI score, acuity, mean zip income, SCBCN membership and distance from treatment center. There were 1,367 patients with complete data for this analysis. Initially, a Cox proportional hazard regression model was conducted. Results indicated a significant association of more recent diagnosis year (*HR*= .937; P<0.001), high intensity induction therapy (*HR*= .695; P<0.001), and higher mean neighborhood income (*HR* = .997; P<0.01) with lower risk of mortality. Conversely, older age at diagnosis (*HR* = 1.025; P<0.001), higher acuity, (WBC Thou/mm^3^, *HR* = 1.002; P<0.001) and higher CCI score (*HR* = 1.117; P<0.001) were significantly associated with higher risk of mortality (see Table 2).

**Table 2.** Cox proportional hazards regression model for all HCA Healthcare data (N = 1367).

	**Hazard Ratio**	**95% CI**	***p*-value**
Diagnosis year	**0.937**	**0.9902, 0.9747**	**< 0.001**
Diagnosis age	**1.025**	**1.0187, 1.0312**	**< 0.001**
Gender Male Female	Ref 1.044	0.8998, 1.2120	0.58
Race White Black Other	Ref 0.907 0.900	0.6688, 1.2129 0.6146, 1.3144	0.51 0.60
Intensity Low High	**Ref** **0.695**	**0.5631, 0.8585**	**< 0.001**
Acuity (WBC - thousands/mm^3^)	**1.002**	**1.0009, 1.0026**	**< 0.001**
Weighted CCI score	**1.117**	**1.0725, 1.1636**	**< 0.001**
Neighborhood income (thousands)	**0.997**	**0.9947, 0.9989**	**0.002**
Specialized center No Yes	Ref 0.859	0.7339, 1.0051	0.058
Distance from center (km)	1.000	0.9995, 1.0003	0.74

When examining the assumption of proportionality, the model indicated a global violation that was driven by acuity and CCI. To address this violation, the model was tested again with a stratification of high and low acuity. Acuity (baseline WBC count) was intended as a surrogate for disease severity, and while acuity measured at baseline would have a larger expected effect at the onset of treatment, it did not maintain a strong effect for longer term risk. The determined threshold for high acuity was WBC > 20/cubic mm.^20^ The high acuity model indicated that more recent diagnosis year, high intensity induction therapy, higher mean zip income were associated with lower risk, whereas, older age at diagnosis and CCI score were associated with higher risk. The low acuity model demonstrated similar results, except that mean zip income was not significantly associated, and SCBCN facilities were associated with lower risk for patients. Both stratified models alleviated the global violation of proportionality over time (see Supplemental Tables), though there were still a variable-level violation in the low acuity model for CCI score.

The propagation of violations in the Cox model prompted an examination of the data using an Accelerated Failure Time (AFT) model, a common alternative model used when proportional hazards assumptions are violated.^21^ The AFT model does not require an assumption of proportional hazards. The results from the AFT model demonstrated similar effects as the Cox regression, specifically with age at diagnosis, acuity at baseline and CCI score being significantly associated with decreased life expectancy, whereas high intensity induction therapy, mean zip income, SCBCN facilities being significantly associated with increased life expectancy (see Table 3).

**Table 3.** Accelerated failure time model for all HCA Healthcare data (N = 1367).

	**Deceleration Factor**	**95% CI**	***p*-value**
Diagnosis year	1.062	0.996, 1.133	0.065
Diagnosis age	**0.958**	**0.949, 0.967**	**< 0.001**
Gender Male Female	Ref 0.910	0.712, 1.164	0.45
Race White Black Other	Ref 1.241 1.230	0.765, 2.011 0.677, 2.231	0.38 0.49
Intensity Low High	Ref **1.640**	**1.162, 2.315**	**0.005**
Acuity (WBC - thousands/mm^3^)	**0.996**	**0.994, 0.998**	**< 0.001**
Weighted CCI score	**0.808**	**0.753, 0.867**	**< 0.001**
Neighborhood income (thousands)	**1.005**	**1.001, 1.009**	**0.002**
Specialized center No Yes	Ref **1.307**	**1.008, 1.696**	**0.044**
Distance from center (km)	1.000	0.999, 1.001	0.84

## Discussion

We conducted a large community-based study with the objective of identifying patient, disease, therapy-intensity and center-related predictors of real-world AML outcomes. The study period between 2011 and 2018 encompassed the introduction of low-intensity hypomethylating-agent based induction, but excluded the more recent developments of targeted therapy using Bcl2, Flt3 and IDH1/2 inhibitors. Our findings support the overall strategy in our community health network for establishment of specialized high-volume centers for the treatment of AML. It is noteworthy that the network could effectively concentrate AML care into these 6 specialized centers treating 799 AML patients versus 592 distributed across the other 63 non-specialized hospitals. For AML patients treated with high intensity chemotherapy induction, initial management at a specialized center produced superior survival. Outcomes were also influenced by year of diagnosis, age, initial WBC count (acuity), neighborhood income, therapeutic intensity, and comorbidity. It is important to note that the survival benefit at specialized centers was not at the cost of inferior outcomes at nonspecialized centers. Median overall survivals for AML across HCA Healthcare community hospitals in aggregate were superior compared to those treated in the larger national registry (SEER). This difference in survival persisted for the non-specialized centers versus SEER.

Center effects are always controversial in the management of high-complexity malignancy. Setting aside the practical necessity of delivering care in the community, studies in the setting of AML have been inconclusive. Those showing benefits from high volume centers, be they NCICC^11^ or academic centers,^10^ tend to suffer from a referral bias. We observed a similar effect with SCBCN subjects having similar acuity but being younger, with lower comorbidity burden and higher neighborhood income than non-specialized hospital patients. With coarsened exact matching, we showed that a “center effect” prevailed only for subjects treated with high intensity induction. The analysis of facility characteristics demonstrated that qualification of a center as a member of SCBCN was associated with improved survival, underscoring the importance of infrastructure, quality systems and volume in achieving improved outcomes. However, aside from the SCBCN designation, our analysis could not identify specific center characteristics.

Our data add to the evidence suggesting that AML induction can be effectively delivered in the community, provided there is speedy access to high-volume centers for allogeneic transplantation, as in our network. Socioeconomic status altering outcomes is an increasingly recognized theme in heme malignancy^8,22^; our study also confirmed an association between neighborhood income and survival.

Our conclusions are strengthened by the large size, community focus, and comprehensive data availability. This is the first community-based evaluation of treatment intensity, and conclusively showed superior survival for those patients capable of receiving higher intensity induction as opposed to single-agent hypomethylating agents. Methodologically, our study used CEM, which may be more powerful than a propensity score. CEM methodology has been shown to conserve sample size while achieving better balance between groups, in comparison to propensity score matching.^23^ Nevertheless, there are several limitations to generalizability. The HCA Healthcare dataset allowed us to separate patients receiving high intensity from less aggressive induction chemotherapy, whereas SEER does not specify the type of chemotherapy each patient received. Patients in the HCA Healthcare dataset prior to 2016 also exist in SEER’s dataset; however, re-identification and removal of those patients is not possible due to SEER’s data use agreement, which prohibits re-identification. Additionally, HCA Healthcare medical records have more complete follow-up data and a longer time frame for follow-up data collection. Retrospective and registry-based studies in AML have other inherent limitations. While cytogenetic information is probably the best guide to disease aggressiveness, this is not feasible in these data sets and most studies utilize the initial WBC count as a surrogate measure of acuity.

The timing of this study occurred in an era when both high-intensity induction chemotherapy or hypomethylating agents were a widely available global standard, at least in Europe. The finding of superior outcomes with higher-intensity therapy may well be generalizable. The impact of specialized centers is more difficult to generalize outside our network, given that infrastructural capability is a continuum, and there is currently no global certification (such as FACT-JACIE for hematopoietic cellular therapy) for acute leukemia. The strong impact of center effects in our study supports the development of such standards.

Novel therapies have emerged after our study period ending in 2018, such as venetoclax, targeted inhibitors (such as Flt3, IDH1/2) as well as immunotherapy (antibodies and immune-effector cell therapy). These are rapidly changing the therapeutic landscape for AML, and we should expect that high-intensity induction therapy will eventually become obsolete. Nevertheless, novel therapies (such as immune effector cells) will also require unique infrastructure, and the importance of center-effects is likely to remain relevant.

In conclusion, our analysis describes real-world outcomes for AML. It shows the existence of a center-effect for specialized centers independent of NCICCC designation, superior outcomes for higher intensity therapy, and that treatment in the broader community hospital setting was not inferior to the SEER dataset. It also validates the impact of year of diagnosis, age, initial WBC count (acuity), neighborhood income, and comorbidity.

### Ethics

The data collected was retrospective and did not require ethics approval or consent.

### Consent for publication

Not applicable

### Availability of data and materials

The datasets collected and/or analyzed during the current study are available from the corresponding author upon reasonable request.

### Competing Interest

The authors of this publication declare no competing financial or other conflicts of interest.

### Author Contribution

M.B., T.C., L.S., C.M., J.H., A.J., and C.F.L. conceived and designed the study; M.B., J.-H.C., T.C., J.C.C., W.B.D., A.E., S.K., S.M., M.B.M., M.R., F.T.S., A.R., V.B., L.S., C.M., J.H., A.J., N.M., and C.F.L. analyzed and interpreted data and reviewed and revised the manuscript; M.B., A.J., and C.F.L. contributed to the writing of the manuscript; M.B. and C.F.L. supervised the study.

## References

[ref-365236] SEER Cancer Statistics Review, 1975-2016.

[ref-365237] Grimwade D., Walker H., Harrison G.. (2001). The predictive value of hierarchical cytogenetic classification in older adults with acute myeloid leukemia (AML): analysis of 1065 patients entered into the United Kingdom Medical Research Council AML11 trial. Blood.

[ref-365238] Juliusson G., Antunovic P., Derolf A.. (2009). Age and acute myeloid leukemia: real world data on decision to treat and outcomes from the Swedish Acute Leukemia Registry. Blood.

[ref-365239] Bhatt V. R., Shostrom V., Giri S.. (2017). Early mortality and overall survival of acute myeloid leukemia based on facility type. Am J Hematol.

[ref-365240] Fathi A.T., Chen Y.B. (2017). The role of FLT3 inhibitors in the treatment of FLT3-mutated acute myeloid leukemia. Eur J Haematol.

[ref-365241] Ma E., Bonthapally V., Chawla A.. (2016). An Evaluation of Treatment Patterns and Outcomes in Elderly Patients Newly Diagnosed With Acute Myeloid Leukemia: A Retrospective Analysis of Electronic Medical Records From US Community Oncology Practices. Clin Lymphoma Myeloma Leuk.

[ref-365242] Ding L., Martin A. E., Canepa E.. (2019). Disparities in Barriers and Facilitators of Acute Leukemia Diagnosis in Young Patients. Blood.

[ref-365243] Knoble N. B., Alderfer M. A., Hossain M. J. (2016). Socioeconomic status (SES) and childhood acute myeloid leukemia (AML) mortality risk: Analysis of SEER data. Cancer Epidemiol.

[ref-365244] Juliusson G., Lazarevic V., Horstedt A.S., Hagberg O., Hoglund M., Swedish Acute Leukemia Registry G. (2012). Acute myeloid leukemia in the real world: why population-based registries are needed. Blood.

[ref-365245] Giri S., Pathak R., Aryal M. R., Karmacharya P., Bhatt V. R., Martin M. G. (2015). Impact of hospital volume on outcomes of patients undergoing chemotherapy for acute myeloid leukemia: a matched cohort study. Blood.

[ref-365246] Ho G., Wun T., Muffly L.. (2018). Decreased early mortality associated with the treatment of acute myeloid leukemia at National Cancer Institute-designated cancer centers in California. Cancer.

[ref-365247] Ho G., Jonas B. A., Li Q., Brunson A., Wun T., Keegan T. H. M. (2017). Early mortality and complications in hospitalized adult Californians with acute myeloid leukaemia. Br J Haematol.

[ref-365248] Freeman A. T., Meyer A. M., Smitherman A. B.. (2016). Statewide geographic variation in outcomes for adults with acute myeloid leukemia in North Carolina. Cancer.

[ref-365249] Thompson M. P., Waters T. M., Kaplan E. K., McKillop C. N., Martin M. G. (2016). Hospital volume and acute myeloid leukemia mortality in Medicare beneficiaries aged 65 years and older. Blood.

[ref-365250] Unger J. M., LeBlanc M., Blanke C. D. (2017). The Effect of Positive SWOG Treatment Trials on Survival of Patients With Cancer in the US Population. JAMA Oncol.

[ref-365251] Kantarjian H. (2016). Acute myeloid leukemia--major progress over four decades and glimpses into the future. Am J Hematol.

[ref-365252] Charlson M. E., Pompei P., Ales K. L., MacKenzie C. R. (1987). A new method of classifying prognostic comorbidity in longitudinal studies: development and validation. J Chronic Dis.

[ref-365253] IRS (2017). https://www.irs.gov/pub/irs-soi/17zpallnoagi.csv.

[ref-365254] Iacus S. M., King G., Porro G. (2012). Causal Inference Without Balance Checking: Coarsened Exact Matching. Political Analysis.

[ref-365255] de Jonge H. J., Valk P. J., de Bont E. S.. (2011). Prognostic impact of white blood cell count in intermediate risk acute myeloid leukemia: relevance of mutated NPM1 and FLT3-ITD. Haematologica.

[ref-365256] Sayehmiri K., Eshraghian M. R., Mohammad K.. (2008). Prognostic factors of survival time after hematopoietic stem cell transplant in acute lymphoblastic leukemia patients: Cox proportional hazard versus accelerated failure time models. J Exp Clin Cancer Res.

[ref-365257] Bona K., Brazauskas R., He N.. (2021). Neighborhood-Poverty and Pediatric Allogeneic Hematopoietic Cell Transplantation Outcomes: A CIBMTR Analysis. Blood.

[ref-365258] Wells A. R., Hamar B., Bradley C.. (2013). Exploring robust methods for evaluating treatment and comparison groups in chronic care management programs. Popul Health Manag.

